# Factors affecting Hemoglobin A1c in the longitudinal study of the Iranian population using mixed quantile regression

**DOI:** 10.1038/s41598-023-36481-x

**Published:** 2023-06-12

**Authors:** Abbas Bahrampour, Saiedeh Haji-Maghsoudi

**Affiliations:** 1 Modeling in Health Research Center, Institute for Futures Studies in Health, Kerman University of Medical Sciences, Kerman, Iran; 2 Department of Biostatistics and Epidemiology, School of Health, Kerman University of Medical Sciences, Kerman, Iran; 3 Griffith University, Brisbane, QLD Australia

**Keywords:** Medical research, Risk factors

## Abstract

Diabetes, a major non-communicable disease, presents challenges for healthcare systems worldwide. Traditional regression models focus on mean effects, but factors can impact the entire distribution of responses over time. Linear mixed quantile regression models (LQMMs) address this issue. A study involving 2791 diabetic patients in Iran explored the relationship between Hemoglobin A1c (HbA1c) levels and factors such as age, sex, body mass index (BMI), disease duration, cholesterol, triglycerides, ischemic heart disease, and treatments (insulin, oral anti-diabetic drugs, and combination). LQMM analysis examined the association between HbA1c and the explanatory variables. Associations between cholesterol, triglycerides, ischemic heart disease (IHD), insulin, oral anti-diabetic drugs (OADs), a combination of OADs and insulin, and HbA1c levels exhibited varying degrees of correlation across all quantiles (p < 0.05), demonstrating a positive effect. While BMI did not display significant effects in the lower quantiles (p > 0.05), it was found to be significant in the higher quantiles (p < 0.05). The impact of disease duration differed between the low and high quantiles (specifically at the quantiles of 5, 50, and 75; p < 0.05). Age was discovered to have an association with HbA1c in the higher quantiles (specifically at the quantiles of 50, 75, and 95; p < 0.05). The findings reveal important associations and shed light on how these relationships may vary across different quantiles and over time. These insights can serve as guidance for devising effective strategies to manage and monitor HbA1c levels.

## Introduction

In recent decades, non-communicable diseases (NCDs) have become a major health concern globally, with diabetes being a leading cause of death among NCDs^1,2^. The prevalence of diabetes has been rapidly increasing due to various factors such as social, economic, demographic, and environmental influences. The number of adults living with diabetes has more than tripled from 151 million in 2000 to 463 million in 2019, and projections indicate further increases to 578 million in 2030 and 700 million in 2045^3^. This rise is primarily attributed to the increasing prevalence of type 2 diabetes, driven by factors such as obesity, unhealthy diets, and sedentary lifestyles^3^. Diabetes is a major health issue in developing countries^4^. The increased prevalence of diabetes and poor diabetes control in developing countries are contributing to the incidence of diabetes-related complications, which are the most common causes of decreased quality of life, morbidity, and mortality^1,4^.

The Middle East and North Africa (MENA) region has witnessed a significant increase in diabetes prevalence, ranking highest globally and projecting further growth by 2030^5^. This upward trend has contributed to a rise in premature heart disease and stroke, necessitating preventive health policies. The IDF has estimated that the MENA region will have the second-highest growth rate in the number of people with diabetes in the world, projected to increase by 96.2% by 2035^6^. Iran, similar to other countries in the region, has witnessed a comparable trend characterized by urbanization, industrialization, and lifestyle changes, leading to an increased prevalence of diabetes. Projections suggest that the population of individuals with diabetes in Iran is projected to reach 9.24 million by the year 2030^7^. Among Iranian adult participants, the annual crude incidence rate of type 2 diabetes (T2D) was 10 per 1000 person-years of follow-up. Additionally, the overall incidence rate of pre-diabetes/T2D among youth was 36.3 per 1000 person-years, equivalent to around 1% each year^8^. The lifetime risk of diabetes was reported approximately 58% for men and 61% for women, at the age of 20 in Iran^9^.

Hemoglobin A1c (HbA1c) has been proposed as a useful tool for screening and diagnosing type 2 diabetes, offering a reflection of glycemia over the previous 3–4 months^10,11^. Its measurement provides valuable information for monitoring blood sugar control and predicting diabetes complications^12,13^. Specifically, a comprehensive analysis revealed that a rise in A1c levels from 6 to 6.5% was associated with a higher occurrence of diabetes over a five-year period, as indicated by a systematic review^14^. Diabetes is associated with various complications, including early onset nephropathy and chronic kidney disease, diabetic retinopathy, and an increased risk of cardiovascular disease. Overweight and obesity are some of the modifiable risk factors that contribute to the rise in diabetes patients, and key lifestyle changes can be considered in prevention plans to address this issue^15^.

Understanding the factors associated with HbA1c levels at different quantiles can contribute to more effective interventions and prevention programs. Quantile regression, a statistical analysis method, allows for a comprehensive examination of the conditional distribution of a response variable, considering a range of explanatory variables. It overcomes the limitations of traditional regression models by providing insights into effects that would otherwise be overlooked. Quantile regression has gained recognition in health and medical studies, offering a realistic framework and eliminating the assumption of normality^16–21^.

Past research has predominantly concentrated on utilizing mean-based mixed regression to assess the impact of various factors on HbA1c, thereby disregarding the possibility of variations across the complete distribution. Previous studies have primarily focused on mean-based mixed regression to determine effect of factors on HbA1c^22,23^, overlooking potential differences across the entire distribution. This study aims to investigate whether the effects of covariates on HbA1c differ across different conditional quantiles (lower and upper tail and middle part). By taking into account the longitudinal nature of the data, this analysis provides a comprehensive understanding of how covariates affect the entire conditional distribution of HbA1c. The findings will contribute to a better understanding of the relationship between factors and HbA1c, offering valuable insights for policy-making and the development of prevention programs.

## Results

**Figure 1:**
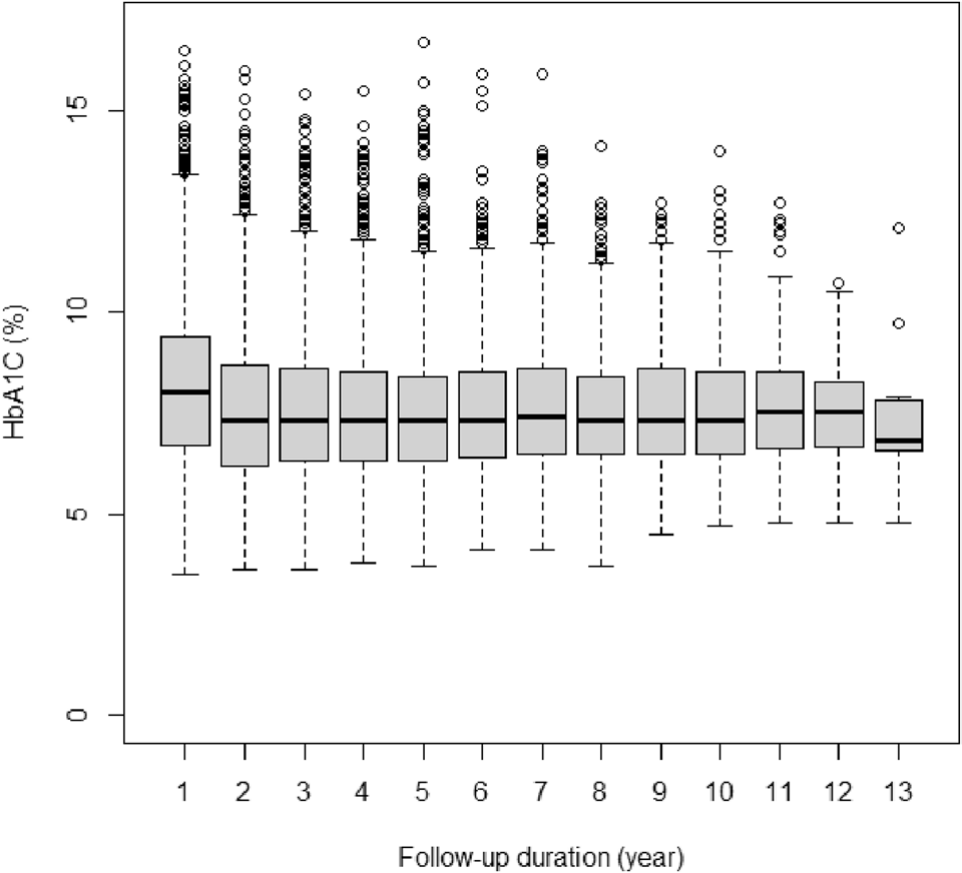
The distribution of HbA1c levels across the follow-up duration.

The study enrolled a cohort of 2791 individuals diagnosed with diabetes, with an average age of 49.65 ± 8.70 years. The minimum age recorded at the beginning of the study was 30 years. The participants had an average disease duration of 4.73 ± 4.57 years. Additionally, the average BMI among the participants was 28.41 ± 4.41. Among them, 1748 (62.6%) were women. Approximately 51% and 60% of the participants had high levels of cholesterol (≥ 200) and triglyceride (≥ 150), respectively. Additionally, 1084 (56.3%) had a history of ischemic heart disease (IHD). Furthermore, 8.7%, 3.7%, and 67.9% were treated with insulin, OADs, and a combination of OADs and insulin, respectively. Overall, 91.3% of the participants did not follow any specific diet regime (Table 1). Figure 1 visually represents the spread of HbA1c levels throughout the duration of the follow-up period. Table 2 provides quantiles of HbA1C levels at different follow-up durations, indicating the range of values observed in a follow-up duration. HbA1C percentiles, such as the 25th, 50th (median), 75th, and 95th, represent cutoffs below which specific percentages of values fall. The results demonstrate how HbA1C changes over time, giving insights into the average blood glucose levels and their distribution across various follow-up periods. The data reveals a slight decrease in median HbA1C values as the follow-up duration increases, indicating an improvement in blood glucose control.

**Table 1:** Baseline characteristics of subjects.

Variables	Frequency	Percent
Sex		
Male	1043	37.4
Female	1748	62.6
Cholesterol (mg/dl)		
< 200	1128	49.3
≥ 200	1160	50.7
Triglyceride (mg/dl)		
< 150	921	40.3
≥ 150	1367	59.7
Insulin therapy		
No	2657	96.3
Yes	101	3.7
Oral antidiabetic drugs		
No	884	32.1
Yes	1874	67.9
Oral antidiabetic drugs plus Insulin		
No	2215	80.3
Yes	543	19.7
Diet		
No	2518	91.3
Yes	240	8.7
Ischemic heart disease		
No	1084	56.3
Yes	843	43.7

**Table 2:** HbA1C levels at different quantiles over a follow-up duration.

Follow-up duration (year)	Quantiles of HbA1C (%)				
	5	25	50	75	95
1	5.2	6.7	8.0	9.4	11.7
2	5.0	6.2	7.3	8.7	11.1
3	5.1	6.3	7.3	8.6	10.8
4	5.2	6.3	7.3	8.5	10.7
5	5.2	6.3	7.3	8.4	10.6
6	5.3	6.4	7.3	8.5	10.4
7	5.4	6.5	7.4	8.6	10.8
8	5.4	6.5	7.3	8.4	10.6
9	5.4	6.5	7.3	8.6	10.5
10	5.5	6.5	7.3	8.5	10.5
11	5.7	6.6	7.5	8.5	10.5
12	5.4	6.7	7.5	8.3	9.9
13	5.5	6.6	6.8	7.8	10.8

The table provides data on HbA1C levels at different quantiles over a follow-up duration of several years. Each row represents a specific year, while the columns represent the quantiles of HbA1C levels: 5th, 25th, 50th (median), 75th, and 95th.

The effects of various influential factors on HbA1c across different quantiles are presented in Table 3. Age demonstrates a significant negative association with HbA1c in the q50, q75, and q95 quantiles (p < 0.001). There is no statistically significant relationship between follow-up time and HbA1c for most quantiles, except for the q25 quantile (p = 0.045). In the q25 quantile, there is a statistically significant positive association, suggesting a slight increase in HbA1c with longer follow-up time. There are no significant associations between sex and HbA1c levels in any of the quantiles. The coefficients are relatively small and not statistically significant, suggesting that sex does not play a substantial role in determining HbA1c. BMI shows a significant positive association with HbA1c in median and higher quantiles (p < 0.001). This indicates that higher BMI values are associated with higher HbA1c levels across the distribution, suggesting a link between body weight and HbA1c. Cholesterol, triglyceride, ischemic heart disease (IHD), insulin, OADs, and the combination of OADs plus insulin demonstrated significant associations across all quantiles (p < 0.001).

**Table 3:** Estimated coefficients and standard errors (SE) of variables across various quantiles of HbA1C.

Variables	q5		q25		q50		q75		q95	
	Coefficient (SE)	p value	Coefficient (SE)	p value	Coefficient (SE)	p value	Coefficient (SE)	p value	Coefficient (SE)	p value
Constant	4.0670 (0.2055)	< 0.001	5.1325 (0.2534)	< 0.001	6.5174 (0.2701)	< 0.001	7.8044 (0.2765)	< 0.001	9.2559 (0.2137)	< 0.001
Follow-up duration (year)	0.0446 (0.0524)	0.395	0.1181 (0.0589)	0.045	0.0597 (0.0620)	0.335	− 0.0947 (0.0609)	0.120	− 0.0327 (0.0431)	0.448
Age (year)	0.0013 (0.0023)	0.586	− 0.0029 (0.0028)	0.301	− 0.0107 (0.0032)	0.001	− 0.0200 (0.0032)	< 0.001	− 0.0303 (0.0024)	< 0.001
Sex (female)	− 0.0345 (0.0425)	0.417	0.0422 (0.0540)	0.434	0.0047 (0.0606)	0.938	− 0.0929 (0.0587)	0.114	− 0.0058 (0.0460)	0.900
BMI (kg/m^2^)	0.0061 (0.0049)	0.211	0.0084 (0.0058)	0.147	0.0233 (0.0062)	< 0.001	0.0262 (0.0064)	< 0.001	0.0231 (0.0047)	< 0.001
Disease duration (year)	0.0105 (0.0037)	0.004	− 0.0016 (0.0049)	0.748	− 0.0115 (0.0054)	0.032	− 0.0126 (0.0055)	0.022	0.0053 (0.0046)	0.242
Chol (mg/dl)	0.3925 (0.0325)	< 0.001	0.4940 (0.0405)	< 0.001	0.4808 (0.0440)	< 0.001	0.6411 (0.0414)	< 0.001	0.7822 (0.0352)	< 0.001
TG (mg/dl)	0.2088 (0.0318)	< 0.001	0.2716 (0.0393)	< 0.001	0.2261 (0.0436)	< 0.001	0.2355 (0.0420)	< 0.001	0.1829 (0.0352)	< 0.001
IHD	0.1411 (0.0380)	< 0.001	0.0987 (0.0475)	0.038	0.1619 (0.0510)	0.002	0.1487 (0.0518)	0.004	0.1801 (0.0424)	< 0.001
Insulin therapy	0.7540 (0.1310)	< 0.001	1.0997 (0.1683)	< 0.001	1.2855 (0.1698)	< 0.001	1.4348 (0.1769)	< 0.001	1.6924 (0.1739)	< 0.001
OADs	0.4865 (0.0739)	< 0.001	0.6488 (0.0867)	< 0.001	0.4608 (0.1086)	< 0.001	0.6122 (0.1065)	< 0.001	1.0480 (0.0820)	< 0.001
OADs plus insulin	0.9996 (0.0817)	< 0.001	1.5725 (0.1001)	< 0.001	1.6636 (0.1170)	< 0.001	1.9412 (0.1188)	< 0.001	2.3278 (0.0923)	< 0.001
Follow-up duration * age	− 0.0003 (0.0005)	0.572	− 0.0011 (0.0006)	0.080	− 0.0005 (0.0007)	0.482	− 0.0005 (0.0007)	0.502	0.0007 (0.0005)	0.198
Follow-up duration * sex	0.0133 (0.0101)	0.187	0.0021 (0.0120)	0.861	0.0074 (0.0129)	0.566	0.0303 (0.0136)	0.026	− 0.0188 (0.0105)	0.073
Follow-up duration * BMI	− 0.0001 (0.0012)	0.964	− 0.0004 (0.0012)	0.776	− 0.0023 (0.0013)	0.071	0.0002 (0.0014)	0.861	0.0049 (0.0010)	< 0.001
Follow−up duration * disease duration	0.0019 (0.0008)	0.021	0.0054 (0.0010)	< 0.001	0.0078 (0.0010)	< 0.001	0.0094 (0.0010)	< 0.001	0.0025 (0.0009)	0.004
Follow-up duration * Chol	− 0.0540 (0.0080)	< 0.001	− 0.0620 (0.0095)	< 0.001	− 0.0592 (0.0100)	< 0.001	− 0.0666 (0.0095)	< 0.001	− 0.0221 (0.0082)	0.007
Follow-up duration * TG	− 0.0106 (0.0075)	0.156	− 0.0073 (0.0086)	0.400	0.0073 (0.0094)	0.434	0.0111 (0.0091)	0.224	0.0331 (0.0077)	< 0.001
Follow-up duration * IHD	− 0.0252 (0.0090)	0.005	− 0.0206 (0.0102)	0.044	− 0.0173 (0.0110)	0.115	− 0.0107 (0.0116)	0.358	− 0.0236 (0.0094)	0.012
Follow-up duration * insulin therapy	0.0015 (0.0292)	0.958	− 0.0601 (0.0391)	0.125	− 0.1360 (0.0409)	0.001	− 0.0901 (0.0439)	0.040	− 0.1003 (0.0337)	0.003
Follow-up duration * OAD	− 0.0074 (0.0161)	0.647	− 0.0301 (0.0212)	0.156	0.0018 (0.0219)	0.935	0.0089 (0.0217)	0.684	− 0.0521 (0.0166)	0.002
Follow-up duration * OADs plus insulin	− 0.0727 (0.0181)	< 0.001	− 0.1346 (0.0237)	< 0.001	− 0.1365 (0.0238)	< 0.001	− 0.1495 (0.0242)	< 0.001	− 0.1633 (0.0184)	< 0.001

*BMI* Body mass index, *Chol* Cholesterol, *TG* Triglyceride, *IHD* Ischemic heart disease, *OADs* Oral anti-diabetic drugs. The table displays the estimated coefficients and standard errors (SE) of different variables across various quantiles, based on linear mixed quantile regression. The dependent variable is HbA1c. Each row corresponds to a specific variable, and each column represents a quantile (q5, q25, q50, q75, and q95). The coefficients reveal the direction and magnitude of the relationship between each variable and HbA1c within each quantile, while the standard errors provide an indication of the precision of the estimates.

The interaction terms between follow-up duration and each variable provide insights into how the relationships evolve over time. In general, the coefficients for the follow-up duration interactions are negative, indicating a decreasing effect over time. However, not all interaction terms are statistically significant. The interaction terms involving follow-up duration and age, sex, and BMI do not consistently show statistically significant associations with HbA1c across the quantiles, except for the third quartile where a significant and noteworthy interaction effect between follow-up duration and sex was observed (p = 0.026). This suggests that, within this specific quantile, the decline in HbA1c levels over time is comparatively less noticeable in females when compared to males.

In the 95th quantile, a statistically significant positive interaction between time and BMI was observed, indicating that the influence of BMI on HbA1c levels becomes increasingly prominent as the follow-up duration progresses. Additionally, across all quantiles, there was a positive interaction between disease duration and follow-up duration, signifying that as the follow-up duration extends, the association between disease duration and HbA1c levels becomes more prominent. Across all quantiles, there was a significant negative interaction effect between cholesterol and follow-up duration. This suggests that the positive impact of cholesterol on HbA1c levels diminishes as the follow-up duration increases. Furthermore, in the 95th quantile, the negative interaction between time and triglyceride signifies a diminishing positive impact of triglyceride over the course of follow-up duration. Additionally, the negative and significant interaction effect between insulin and follow-up duration suggests that the positive influence of insulin on HbA1c decreases as follow-up duration progresses in the middle and higher quantiles. Based on the negative interaction effect between OADs and follow-up duration, the positive impact of OADs diminishes as time progresses in the 95th quantile. Additionally, the interaction effect between IHD and follow-up duration was negative in the 5th, 25th, and 95th quantiles, indicating a declining positive effect of IHD on HbA1c over time. Furthermore, across all quantiles, the interaction effect between follow-up duration and OADs plus insulin was significantly negative, implying a decreasing positive effect of OADs plus insulin on HbA1c as follow-up duration increases (Table 3).

**Figure 2:**
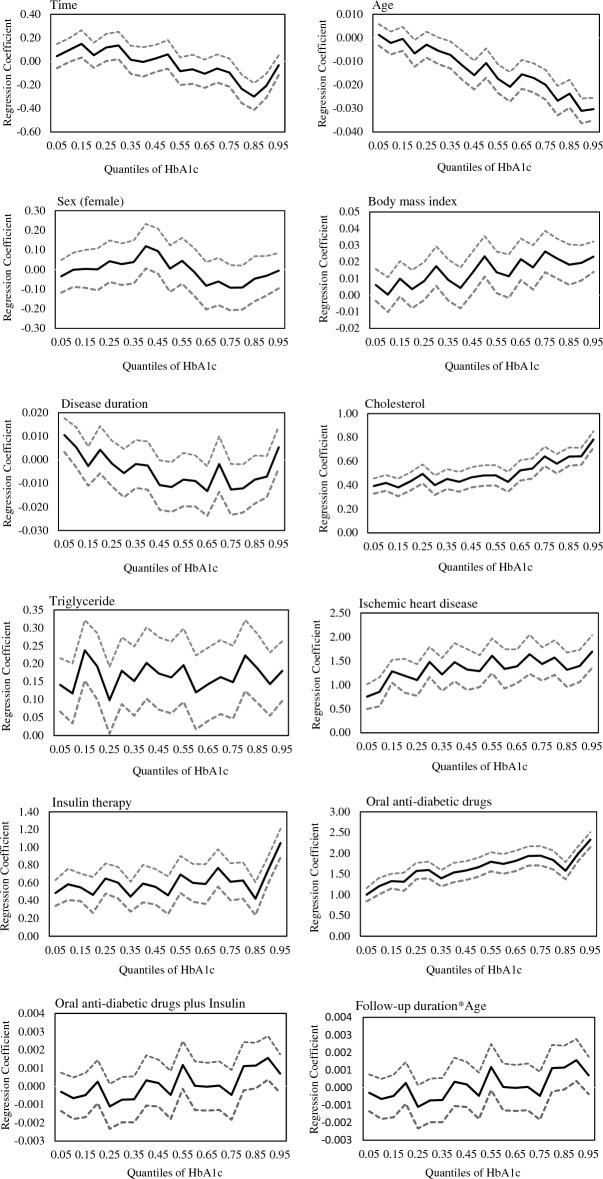

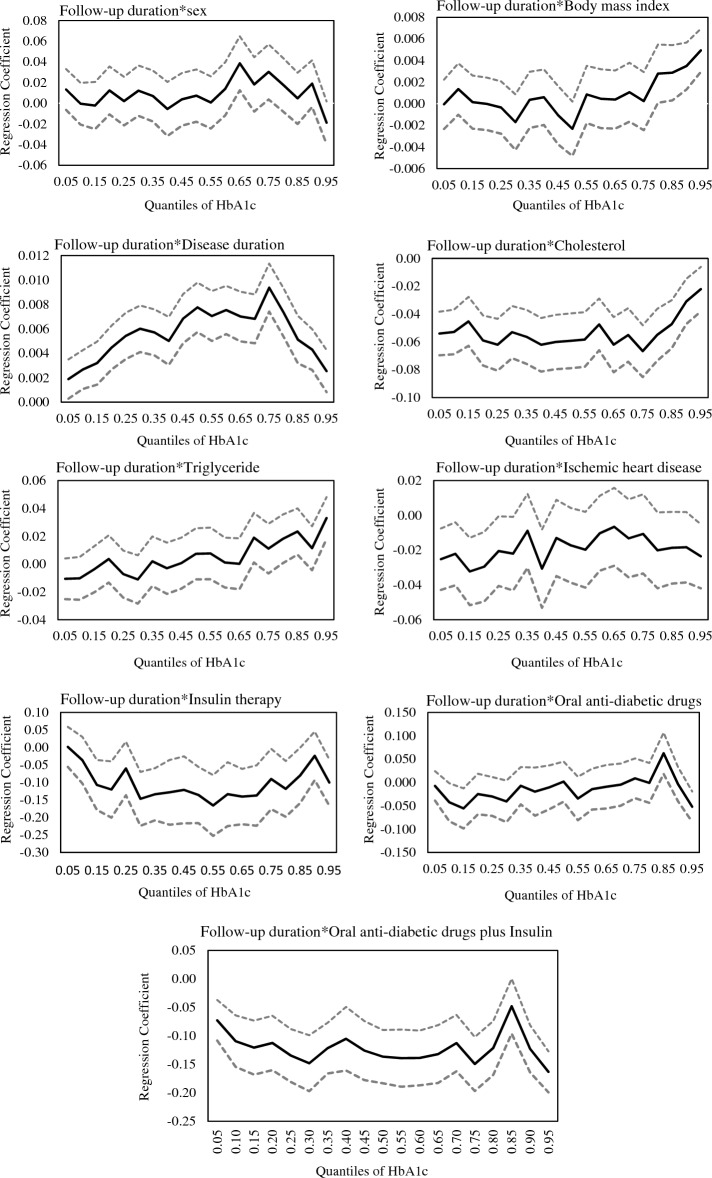
Estimated effects (solid line) and 95% confidence intervals (dashed lines) of various variables on HbA1C across different quantiles.

The effect of all variables on HbA1c across different quantiles is depicted in Fig. 2. According to this figure, the impact of variables varies across quantiles of HbA1c. The declining effect of age exhibited an increasing trend over time, particularly pronounced at higher levels of HbA1c. Both BMI and triglyceride showed increasing effects over time, with greater increases observed at elevated HbA1c levels. Conversely, the effects of IHD, OADs, OADs plus insulin, and insulin demonstrated a decreasing trend over time, and this decline was more prominent at higher levels of HbA1c. The increasing effect of cholesterol diminished over time, especially noticeable at lower HbA1c levels. Additionally, the disease duration had a substantial impact on both low and high HbA1c levels, but its influence weakened over time in the upper and lower percentiles compared to changes in the middle percentiles.

## Discussion

The analysis of linear mixed quantile regression has yielded valuable insights into the relationships between various variables and HbA1c levels across different quantiles. These findings contribute to our understanding of the strength, direction, and potential changes in these relationships over time. Initially, the analysis revealed a significant negative association between age and HbA1c levels in the q50, q75, and q95 quantiles. These findings suggest that as individuals age, higher quantiles of HbA1c tend to decrease. However, it is important to note that age alone may not be the sole determining factor, and other factors such as lifestyle, diet, and genetic predisposition should also be taken into account. Regarding gender, no significant connections between sex and HbA1c levels across the quantiles were identified in the analysis. The small coefficients lacking statistical significance indicate that sex does not play a substantial role in determining HbA1c levels. This suggests that both men and women may have similar HbA1c levels, highlighting the need to examine other factors when assessing and managing HbA1c levels.

BMI demonstrated a significant positive correlation with HbA1c across all quantiles. This finding indicates that higher BMI values are associated with higher HbA1c levels across the entire distribution. The link between body weight and HbA1c suggests that weight management and maintaining a healthy BMI could potentially help in managing blood sugar levels and reducing the risk of developing metabolic disorders.

Furthermore, the study findings highlight the significance of cholesterol, triglyceride, ischemic heart disease (IHD), insulin, oral antidiabetic medications (OADs), and the combination of OADs plus insulin in relation to HbA1c levels. These associations were observed across all quantiles, indicating their relevance in managing glycemic control in individuals with diabetes. These results emphasize the importance of comprehensive management strategies that address lipid profiles, cardiovascular health, and appropriate medication therapies to achieve optimal glycemic control and improve overall health outcomes in individuals with diabetes. Lipid abnormalities commonly occur in individuals with diabetes mellitus due to insulin resistance and related complications^24^. Achieving optimal glycemic control and improving insulin sensitivity are crucial in mitigating the risk of diabetes-related complications^25^. Several studies have reported significant associations between cholesterol and triglyceride levels with HbA1c levels. A longitudinal study reported significant associations between cholesterol and triglyceride levels with HbA1c levels^23^. Furthermore, another cross-sectional study observed a positive association between triglyceride levels and HbA1c^26^. Additionally, another study found a positive association between HbA1c and high triglyceride levels^27^. Furthermore, the results of other studies indicate patients with poor glycemic control demonstrated higher levels of total cholesterol, low-density triglycerides. These lipid profile findings were significantly correlated with HbA1c levels.

In one cross-sectional study, it was found that total cholesterol was not significant in good glycemic ‌control (HbA1c < 7.0%) and poor glycemic control (HbA1c > 7.0%)^25^. Discrepancy in this finding may stem from dissimilarities in the characteristics of the study populations and variations in the analytical approaches utilized.

Managing weight is essential in preventing diabetes mellitus (DM), particularly in relation to abdominal obesity^28–30^. Obesity can impair insulin sensitivity by damaging insulin receptors and β-cell function in the pancreas^31,32^.

A specific retrospective cross-sectional study, the results demonstrated a significant positive association between HbA1c and TG levels based on the correlation coefficient. Additionally, their study utilized linear regression analysis, which indicated that HbA1c values were associated with TG levels and were independent of factors such as age, BMI, total cholesterol, and fasting blood glucose (FBG) levels^33^. In a separate investigation, it was noted that among males, it was observed that for males, there was a positive association between BMI and FBG across a wide range of values. However, this relationship weakened as the regression coefficient declined. Age consistently displayed a positive association with FBG across all quantiles, showing a slight upward trend. TG also showed a positive correlation with FBG in males. Similarly, for females, BMI showed a positive relationship with FBG in the lower and middle quantiles. Age displayed a consistent and increasing positive association with FBG across all quantiles in females as well^34^. However, in higher quantiles, the effect of age in our study was negative, which contradicts the findings of a previous study. This inconsistency may be attributed to differences in the characteristics of the study populations. Our study focused specifically on diabetic patients, whereas the mentioned study encompassed the general population. Furthermore, the outcomes can be influenced by factors such as the duration of the disease and the treatments administered.

One study indicated a connection between higher HbA1c levels and increased BMI in childhood and adulthood. Greater BMI gains during early life were associated with a higher likelihood of HbA1c exceeding 7% later in adulthood. While the relationship between BMI gains in adulthood and HbA1c levels was influenced by the achieved BMI, it remained significant over a long period, indicating an independent effect^35^. Previous research has also highlighted the relationship between obesity and impaired blood sugar control^36^. Furthermore, our findings align with previous studies that have demonstrated positive associations between HbA1c and cardiovascular disease in individuals with diabetes^37^. These connections emphasize the importance of monitoring and managing HbA1c levels in relation to cardiovascular health.

In general, these findings differentiate risk factors across the spectrum of low to high HbA1c levels. In certain scenarios, it is crucial to differentiate individuals with diabetes who have extremely low or high HbA1c levels from those with intermediate levels. This distinction is essential for identifying influential factors for low, intermediate, and high values of HbA1c in diabetes management. Previous studies have employed linear mixed models for this purpose^22,23^, but these models were constrained in their ability to elucidate the effects of factors on the entire distribution of the response variable. However, it is vital to acknowledge the limitations of this study and take into account the specific characteristics of the population being studied when interpreting these findings. Further research and validation studies are necessary to confirm and expand upon these results, as well as to explore potential underlying mechanisms contributing to the observed associations. Moreover, in this study, we did not provide explicit information regarding the dietary patterns of the participants. Additionally, we did not investigate the mental and emotional state of the individuals, which has been recognized in previous research as an influential factor affecting high levels of fasting blood sugar (FBS) and HbA1c. Furthermore, the role of genetic factors, which are also significant, was not assessed. Future research endeavors should consider evaluating these factors to gain deeper insights and develop a more comprehensive understanding of their impact on FBS and HbA1c levels.

In conclusion, the results of our study underscore the relationships between age, cholesterol, triglyceride, ischemic heart disease, insulin, OADs, and OADs plus insulin with HbA1c levels across different quantiles. BMI is positively associated with high HbA1c levels. These findings highlight the importance of considering these factors in diabetes management and emphasize the importance of recognizing the differential impact of risk factors on patients with different HbA1c levels for both public health planning and patient assessment.

## Methods

A retrospective study based on a sample of 2791 with diabetes who had been referred to Isfahan Endocrine and Metabolism Research Center during 2000–2012 was conducted. Characteristic information of individuals registered in this center, who have Type 2 Diabetes Mellitus (T2DM), was extracted from the center's database^22^. This study exclusively included subjects who had a minimum of three measurements. The patients included in the study were those who had been diagnosed with type 2 diabetes and were aged 30 years or older. They were required to have at least three measurements taken, with one measurement recorded each year. Each measurement documented the HbA1c test results. To identify individuals with diabetes, the criteria used were in accordance with the American Diabetes Association guidelines, which included fasting plasma glucose levels of 126 mg/dl or higher, an oral glucose tolerance test result of 200 mg/dl or higher, random glucose levels of 200 mg/dl or higher, and the presence of symptoms^38^.

## Outcome and covariates

The central objective was to evaluate HbA1c levels as a primary outcome of interest. The variables of age (in years), sex, body mass index (BMI in kg/m^2^), disease duration (in years), cholesterol (categorized as less than 200 mg/dl as normal values and equal to or greater than 200 mg/dl)^39,40^, and triglyceride (categorized as less than 150 mg/dl as normal levels and equal to or greater than 150 mg/dl)^41^, as well as ischemic heart disease (IHD), also known as chronic coronary syndrome, were included as covariates. Furthermore, insulin therapy, oral anti-diabetic drugs (OADs) (including all antidiabetic drugs), and the combination of OADs and insulin were included as additional covariates in the model.

BMI was determined by dividing the weight in kilograms by the square of the height in meters. The measurements were taken while patients were wearing light clothing and no footwear, utilizing a Seca stadiometer^42^. Blood samples were obtained from all participants after a period of fasting for 10–12 h. The levels of lipid profile were determined using an enzymatic colorimetric method (ParsAzmoon, Tehran, Iran) on a Selectra-2 auto-analyzer (Vital Scientific, Spankeren, Netherlands), which was specifically adapted for this analysis^43^. The duration of diabetes was calculated from the baseline examination, estimated based on the first abnormal laboratory report or the date of diabetes-related treatment, and tracked throughout the follow-up period. BMI and sex were regarded as time-constant variables, based on the baseline measurement, while the other variables were considered as time-varying. Although age and disease duration were initially measured at baseline, for each subsequent year of follow-up, the baseline values were incremented by one.

### Statistical methods

In longitudinal studies, multiple measurements are gathered for individuals over a period of time. The main objective is often to identify changes in the response over time and the associated factors that influence these changes. The effects of these factors can be associated with both the overall level and the pattern of the response distribution over time. The study utilized the linear quantile mixed models (LQMM)^44^ to examine the impact of covariates on the response at different quantiles of the HbA1c distribution. LQMM allows for the inclusion of both random effects and fixed effects in the model, serving as an extension of both quantile regression (QR) and linear mixed models. LQMM offers flexibility in investigating the effects of covariates on the entire conditional distribution of the response, while also accounting for the dependency between measurements from the same subjects. In the LQMMs, the quantiles of the outcome variable were modeled as a function of the covariates. The model is represented in the following manner:

$$\begin{aligned} {{\varvec{\mu}}}_{{\varvec{i}}}^{{\varvec{\tau}}}&={{\varvec{X}}}_{{\varvec{i}}}{{\varvec{\beta}}}_{{\varvec{x}}}^{({\varvec{\tau}})}+{{\varvec{Z}}}_{{\varvec{i}}}{{\varvec{u}}}_{{\varvec{i}}},\\ & \quad i=1, 2 \ldots, n\end{aligned}$$

where ${{\varvec{\beta}}}_{{\varvec{x}}}^{({\varvec{\tau}})}$ is a vector of unknown fixed effects in the *τ*th quantile. Furthermore, ***u***_i_ is a *p*-dimension vector of random effects. It is assumed the ***u***_i_ to be a zero-median random vector. It is independent from the model’s error term. Also, ***X***_i_ and ***Z***_i_ represent the design matrix of fixed and random effects of subject *i*, respectively.

The matrix formulation of *τ*th LQMM is given by:

$${\varvec{y}}={{\varvec{\mu}}}^{({\varvec{\tau}})}+{{\varvec{\varepsilon}}}^{({\varvec{\tau}})},$$

where:

$${{\varvec{\mu}}}^{({\varvec{\tau}})}={{\varvec{X}}}_{{\varvec{i}}}{{\varvec{\beta}}}^{({\varvec{\tau}})}+{\varvec{Z}}{\varvec{u}}.$$

The data analysis was performed using R software version 4.1.2^45^. The significance level was set at a p value of less than 0.05 to determine statistical significance.

### Ethical approval and consent to participate

The data used in this study were secondary data and there is no access to patients' names to obtain consent. A waiver of informed consent was awarded for the analyses conducted in this study by the ethics committee of Kerman University of Medical Sciences. All methods were carried out in accordance with relevant guidelines. This study was approved by Ethics Committee of the Kerman University of Medical Sciences. The ethical code for this study is IR.KMU.REC.1401.500.

## Data Availability

The data that support the findings of this study are available from Abbas Bahrampour (Email: a_bahrampour@kmu.ac.ir) but restrictions apply to the availability of these data, which were used under license for the current study, and so are not publicly available. Data are however available from the authors upon reasonable request and with permission of the first author.

## Abbreviations

HbA1c: Hemoglobin A1c
LQMMs: Linear mixed quantile regression models
BMI: Body mass index
IHD: Ischemic heart disease
OADs: Oral anti-diabetic drugs
NCDs: Non-communicable diseases
IDF: International Diabetes Federation
MENA: Middle East and North Africa
DALY: Disability-adjusted life year

## References

[1] Harding JL, Pavkov ME, Magliano DJ, Shaw JE, Gregg EW (2019). Global trends in diabetes complications: A review of current evidence. Diabetologia.

[2] Peykari N (2015). Diabetes research in Middle East countries; a scientometrics study from 1990 to 2012. J. Res. Med. Sci..

[3] International Diabetes Federation. IDF Diabetes Atlas, 9th edn. Brussels: International Diabetes Federation, 2019. https://diabetesatlas.org/en/sections/worldwide-toll-of-diabetes.html. Accessed 17 Oct 2021.

[4] Abrishami Z, Nasli-Esfahani E, Razmandeh R, Larijani B, Bandarian F (2017). Iran diabetes research roadmap (IDRR) study; gap analysis of diabetes complications in Iran: A review article. Iran. J. Public Health.

[5] Noshad S, Afarideh M, Heidari B, Mechanick JI, Esteghamati A (2015). Diabetes care in Iran: Where we stand and where we are headed. Ann. Glob. Health.

[6] Guariguata L (2014). Global estimates of diabetes prevalence for 2013 and projections for 2035. Diabetes Res. Clin. Pract..

[7] Javanbakht M, Mashayekhi A, Baradaran HR, Haghdoost A, Afshin A (2015). Projection of diabetes population size and associated economic burden through 2030 in Iran: Evidence from micro-simulation Markov model and Bayesian meta-analysis. PLoS One.

[8] Ramezankhani A (2018). Diabetes mellitus: Findings from 20 years of the Tehran lipid and glucose study. Int. J. Endocrinol. Metab..

[9] Ramezankhani A, Habibi-Moeini AS, Zadeh SST, Azizi F, Hadaegh F (2022). Effect of family history of diabetes and obesity status on lifetime risk of type 2 diabetes in the Iranian population. J. Glob. Health.

[10] Kidwai SS, Nageen A, Bashir F, Ara J (2020). HbA1c—a predictor of dyslipidemia in type 2 Diabetes Mellitus. Pak. J. Med. Sci..

[11] Kramer CK, Araneta MRG, Barrett-Connor E (2010). A1C and diabetes diagnosis: The Rancho Bernardo Study. Diabetes Care.

[12] Gallagher EJ, Le Roith D, Bloomgarden Z (2009). Review of hemoglobin A1c in the management of diabetes. J. Diabetes.

[13] Selvin E (2021). Hemoglobin A1c—using epidemiology to guide medical practice: Kelly West Award lecture 2020. Diabetes Care.

[14] Association AD (2016). 2 Classification and diagnosis of diabetes. Diabetes Care.

[15] Al-Rifai RH (2019). Type 2 diabetes and pre-diabetes mellitus: A systematic review and meta-analysis of prevalence studies in women of childbearing age in the Middle East and North Africa, 2000–2018. Syst. Rev..

[16] Gebregziabher M (2011). Using quantile regression to investigate racial disparities in medication non-adherence. BMC Med. Res. Methodol..

[17] Geraci M (2014). Linear quantile mixed models: The lqmm package for Laplace quantile regression. J. Stat. Softw..

[18] Jang Y, Kim JH, Lee H, Lee K, Ahn SH (2018). A quantile regression approach to explain the relationship of Fatigue and Cortisol, Cytokine among Koreans with Hepatitis B. Sci. Rep..

[19] Gebremariam MK (2018). Change in BMI distribution over a 24-year period and associated socioeconomic gradients: A quantile regression analysis. Obesity.

[20] Lamichhane DK (2020). Quantile regression analysis of the socioeconomic inequalities in air pollution and birth weight. Environ. Int..

[21] Kim MY, Lee EJ (2019). Factors affecting self-care behavior levels among elderly patients with type 2 diabetes: A quantile regression approach. Medicina.

[22] Kazemi E, Hosseini SM, Bahrampour A, Faghihimani E, Amini M (2014). Predicting of trend of hemoglobin a1c in type 2 diabetes: A longitudinal linear mixed model. Int. J. Prev. Med..

[23] Jalali M, Shahraki HR, Bahrampour A, Ayatollahi SMT (2017). Application of penalized mixed model in identification of most associated factors with hemoglobin A1c level in Type 2 diabetes. Glob. J. Health Sci..

[24] Ormazabal V (2018). Association between insulin resistance and the development of cardiovascular disease. Cardiovasc. Diabetol..

[25] Selvi NMK (2021). Association of triglyceride-glucose index (TyG index) with HbA1c and insulin resistance in type 2 diabetes mellitus. Maedica.

[26] VinodMahato R (2011). Association between glycaemic control and serum lipid profile in type 2 diabetic patients: Glycated haemoglobin as a dual biomarker. Biomed. Res..

[27] Naqvi S (2017). Correlation between glycated hemoglobin and triglyceride level in type 2 diabetes mellitus. Cureus.

[28] Yin M, Augustin B, Shu C, Qin T, Yin P (2016). Probit models to investigate prevalence of total diagnosed and undiagnosed diabetes among aged 45 years or older adults in China. PLoS One.

[29] Wysocka-Mincewicz M, Kołodziejczyk H, Wierzbicka E, Szalecki M (2015). Overweight, obesity and lipids abnormalities in adolescents with type 1 diabetes. Pediatr. Endocrinol. Diabetes Metab..

[30] McKeigue P, Shah B, Marmot M (1991). Relation of central obesity and insulin resistance with high diabetes prevalence and cardiovascular risk in South Asians. Lancet.

[31] Roth CL, Reinehr T (2010). Roles of gastrointestinal and adipose tissue peptides in childhood obesity and changes after weight loss due to lifestyle intervention. Arch. Pediatr. Adolesc. Med..

[32] Kahn SE, Hull RL, Utzschneider KM (2006). Mechanisms linking obesity to insulin resistance and type 2 diabetes. Nature.

[33] Alzahrani SH (2019). Association between glycated hemoglobin (HbA1c) and the lipid profile in patients with type 2 diabetes mellitus at a tertiary care hospital: A retrospective study. Diabetes Metab. Syndrome Obes. Targets Ther..

[34] Guo X (2017). Associations of fasting blood glucose with influencing factors in Northeast China: A quantile regression analysis. Int. J. Environ. Res. Public Health.

[35] Power C, Thomas C (2011). Changes in BMI, duration of overweight and obesity, and glucose metabolism: 45 years of follow-up of a birth cohort. Diabetes Care.

[36] Firouzi S, Barakatun-Nisak MY, Azmi KN (2015). Nutritional status, glycemic control and its associated risk factors among a sample of type 2 diabetic individuals, a pilot study. J. Res. Med. Sci..

[37] Lee SW (2017). Association between HbA1c and carotid atherosclerosis among elderly Koreans with normal fasting glucose. PLoS One.

[38] Association AD (2013). Standards of medical care in diabetes—2013. Diabetes Care.

[39] Fernández-Friera L (2017). Normal LDL-cholesterol levels are associated with subclinical atherosclerosis in the absence of risk factors. J. Am. Coll. Cardiol..

[40] Thomas F (2002). Combined effects of systolic blood pressure and serum cholesterol on cardiovascular mortality in young (< 55 years) men and women. Eur. Heart J..

[41] Klempfner R (2016). Elevated triglyceride level is independently associated with increased all-cause mortality in patients with established coronary heart disease: Twenty-two-year follow-up of the bezafibrate infarction prevention study and registry. Circ. Cardiovasc. Qual. Outcomes.

[42] Salehidoost R, Mansouri A, Amini M, Aminorroaya Yamini S, Aminorroaya A (2020). Diabetes and all-cause mortality, a 18-year follow-up study. Sci. Rep..

[43] Safari S, Abdoli M, Amini M, Aminorroaya A, Feizi A (2021). A 16-year prospective cohort study to evaluate effects of long-term fluctuations in obesity indices of prediabetics on the incidence of future diabetes. Sci. Rep..

[44] Galarza CE, Lachos VH, Bandyopadhyay D (2017). Quantile regression in linear mixed models: A stochastic approximation EM approach. Stat. Interface.

[45] Team, R. C. (2021).

